# Protocol for Past BP: a randomised controlled trial of different blood pressure targets for people with a history of stroke of transient ischaemic attack (TIA) in primary care

**DOI:** 10.1186/1471-2261-10-37

**Published:** 2010-08-09

**Authors:** Kate Fletcher, Jonathan Mant, Richard McManus, Sarah Campbell, Jonathan Betts, Clare Taylor, Satnam Virdee, Sue Jowett, Una Martin, Sheila Greenfield, Gary Ford, Nick Freemantle, FD Richard Hobbs

**Affiliations:** 1 Primary Care Clinical Sciences, Clinical Sciences Building University of Birmingham, Edgbaston Birmingham UK, B15 2TT; 2 General Practice & Primary Care Research Unit, Department of Public Health & Primary Care, University of Cambridge, UK, CB2 0SR; 3 Clinical Research facility Royal Victoria Infirmary Newcastle Upon Tyne UK, NE1 4LP

## Abstract

**Background:**

Blood pressure (BP) lowering in people who have had a stroke or transient ischaemic attack (TIA) leads to reduced risk of further stroke. However, it is not clear what the target BP should be, since intensification of therapy may lead to additional adverse effects. PAST BP will determine whether more intensive BP targets can be achieved in a primary care setting, and whether more intensive therapy is associated with adverse effects on quality of life.

**Methods/Design:**

This is a randomised controlled trial (RCT) in patients with a past history of stroke or TIA. Patients will be randomised to two groups and will either have their blood pressure (BP) lowered intensively to a target of 130 mmHg systolic, (or by 10 mmHg if the baseline systolic pressure is between 125 and 140 mmHg) compared to a standard group where the BP will be reduced to a target of 140 mmHg systolic. Patients will be managed by their practice at 1-3 month intervals depending on level of BP and followed-up by the research team at six monthly intervals for 12 months.

610 patients will be recruited from approximately 50 general practices. The following exclusion criteria will be applied: systolic BP <125 mmHg at baseline, 3 or more anti-hypertensive agents, orthostatic hypotension, diabetes mellitus with microalbuminuria or other condition requiring a lower treatment target or terminal illness.

The primary outcome will be change in systolic BP over twelve months. Secondary outcomes include quality of life, adverse events and cardiovascular events.

In-depth interviews with 30 patients and 20 health care practitioners will be undertaken to investigate patient and healthcare professionals understanding and views of BP management.

**Discussion:**

The results of this trial will inform whether intensive blood pressure targets can be achieved in people who have had a stroke or TIA in primary care, and help determine whether or not further research is required before recommending such targets for this population.

**Trial Registration:**

ISRCTN29062286

## Background

Stroke is the third largest cause of death in England, and the single largest cause of adult disability[1]. A National Audit Office report (2005) highlighted the high cost of stroke to the NHS: about £2.8 billion per year in direct care costs, and an additional £1.8 billion per year cost to the wider economy due to lost productivity and disability [2]. Recent estimates suggest that between 30-45% of strokes are recurrent events, [3] so more effective secondary prevention could result in significant savings: the National Audit Office estimates that preventing just 2% of strokes in England in a year could save care costs of over £37 million [2]. NICE has identified better control of hypertension as one of the interventions that are cost saving for the NHS [4].

The PROGRESS trial demonstrated that blood pressure lowering is beneficial in reducing risk of stroke amongst both hypertensive and non-hypertensive individuals with a history of stroke or TIA recruited in secondary care immediately after their cerebrovascular event. In this trial, patients were randomised to either a combination of an ACE inhibitor and thiazide diuretic against double placebo, or an ACE inhibitor alone against single placebo. The decision to randomise to one or two agents was made by the supervising physician on the basis of whether or not they thought it was safe to randomise an individual patient to two agents. Mean blood pressure in the intervention arm was reduced from 147 mmHg systolic by 9 mmHg (SE 0.3), and this was associated with a 28% reduction in stroke risk [5]. The positive result of the PROGRESS trial raises a supplementary question: by how much should blood pressure be lowered? No randomised trials have specifically compared different target blood pressures (BP) in the post-stroke/TIA population. Observational data (although not collected specifically in people with a history of stroke or TIA) suggest that the lower the blood pressure, the lower the risk of vascular mortality, at least down to 115 mmHg systolic [6,7]. There is some evidence from PROGRESS to support this, in that the sub-group of patients whose baseline BP was between 120 and 140 mmHg who were randomised to combination therapy had a significantly reduced risk of stroke compared with control, though this benefit was not observed in patients who were randomised to a single agent [8]. Guidelines have tended to interpret this evidence by recommending a target of 130 mmHg for systolic blood pressure in people with cerebrovascular disease [9,10]. However, the question remains whether such a target is prudent in general (42% were randomised to a single agent and gained no benefit) and whether it is achievable in primary care (PROGRESS was secondary care based) [5].

Long term management of blood pressure following stroke and TIA is predominantly carried out in primary care. Recent studies of blood pressure control in this setting paint a mixed picture of implementation of guidelines. In a study of seven general practices in South Birmingham in 2002, 63% of patients with a previous stroke or TIA had BP above the 140 mmHg target, and 80% above the 130 mmHg target [11]. 68% of these patients were prescribed BP lowering therapy. An analysis of general practice data on the QRESEARCH database for 2002-2004 found that of all patients with incident stroke, blood pressure was not recorded in 25% of patients, and where it was recorded, it was above the 140 mmHg target in 47% [12]. An analysis of the impact of the Quality Outcomes Framework (QOF) carried out for the National Audit Office found that the proportion of people with a history of stroke or TIA who had their BP measured in the preceding 15 months rose from 89% to 95% between 2004 and 2005, and the proportion with a BP below 150 mmHg (the target level for the QOF) rose from 69% to 80% suggesting some improvement [13]. An analysis of the care of over three thousand patients who had a TIA during 2004-5 found that 60% had a BP equal to or below the 140 mmHg target, though only 50% were on any blood pressure lowering therapy [14].

In summary, although there is some evidence that blood pressure lowering in people who have had a stroke or TIA is beneficial, there is no clear guidance on what the target BP should be. Furthermore, data collected from primary care suggest that guidelines from the British Hypertension Society and Intercollegiate Stroke Working Party are not being fully implemented. This research is designed to support implementation of the guidelines by both addressing the gaps in the underlying evidence base, and testing a specific mechanism for implementation of blood pressure lowering.

## Methods/Design

### Study aims

The primary aim of Past BP is to determine whether a more intensive target BP for people with stroke or TIA in a pragmatic primary care setting will lead to a lower BP.

Secondary aims of the research are to:

• determine the impact of a more intensive BP target on patient quality of life;

• identify the barriers to implementation of more intensive blood pressure lowering;

• to explore whether the potential benefits associated with intensive blood pressure lowering might be outweighed by potential adverse effects on quality of life and costs.

The latter point will be explored by economic modelling. If there is uncertainty as to whether there is net benefit from intensive blood pressure lowering, then there would be a case for a trial of different targets for BP lowering in primary care that is powered to detect differences in clinical end-points.

The study will also investigate patients' understanding and beliefs about the relationship between blood pressure and stroke, and patients and healthcare professionals experience of participating in the study, which may contribute to the success or otherwise of the intervention.

### Study design and setting

Past BP is a primary care based pragmatic randomised controlled trial (RCT) in which people with stroke or TIA are randomised to an intensive blood pressure (BP) target group (target 130 mmHg systolic, or 10 mmHg reduction in systolic BP if baseline BP 125 - 140 mmHg) or a standard BP target group (target 140 mmHg systolic).

We will also use qualitative methodologies to investigate patient and healthcare professionals understanding and views of BP management. Grounded theory methods will guide sampling, data collection and data analysis [15,16]. Sampling will be done purposively to allow for the maximum variety of characteristics. Semi-structured interviews will be carried out [17] and will continue until new concepts are no longer being generated and theoretical saturation is reached.

#### Ethical Considerations

Full ethical approval for this study has been obtained from Warwickshire Research Ethics Committee, reference 08/H1211/121. A Data Monitoring Committee and a Trial Steering Committee will monitor the progress of the RCT.

### Randomised controlled trial

#### Study Interventions

Management of both treatment groups will follow study specific treatment protocols that reflect the current NICE guidelines [18]. However, the thresholds for intervention between the two treatment groups are different: all patients in the intensive target arm will have their BP lowering therapy intensified at trial entry since the target will be automatically below their baseline BP whereas only those patients in the standard arm whose BP is above 140/90 mmHg will have their therapy intensified at the outset.

#### Identification of eligible patients

Eligible patients will be identified from general practices from the Central England Primary Care Research Network and from the Midlands Research Practice Consortium (MidReC). Each practice will run a search of their clinical computer system to identify all patients on the stroke/TIA register. Where possible, the computer search will exclude patients with clear exclusion criteria (see table 1). The GP will also remove patients for whom a study invitation would be inappropriate (for example, those with a terminal illness). Patients with no clear exclusion criteria at this stage will be sent a letter inviting them to attend a study baseline clinic appointment.

**Table 1:** Study inclusion and exclusion criteria

*Inclusion criteria*
On practice TIA/stroke register

***Exclusion criteria***

Systolic BP < 125 mmHg at baseline;
Already taking 3 or more anti-hypertensive agents; orthostatic hypotension (>20 mmHg postural change in systolic BP after 1 minute standing)
Patient already has a treatment target of 130 mmHg systolic BP specified
Unable to provide informed consent.
Insufficient corroborative evidence of stroke/TIA from medical record and patient interview

#### Baseline clinic appointment

This clinic appointment is carried out by a Research Nurse. At this appointment the nurse will: confirm the stroke/TIA diagnosis through review of medical records and patient interview; determine whether there are any exclusion criteria present; and collect baseline data (see table 2). If the patient is eligible and willing to take part, the nurse will also gain written informed consent prior to randomisation, and will telephone the randomisation service to obtain treatment group allocation.

**Table 2:** Timing and content of study assessments.

*Baseline data: research nurse administered*
*Socio-demographic characteristics:*
Age; Ethnicity; Gender; Postcode

*Validation of stroke/transient ischaemic attack:*
Review of medical records with patient history

*Clinical measures:*
Six blood pressure (BP) measurements, calculating mean of 2^nd ^and 3^rd ^measurements and recording details of: arm used; arm circumference; BP cuff size; and time BP measurement started
24 hour ambulatory BP recording

*Medical history*
Previous history of angina, myocardial infarction, heart failure, atrial fibrillation, CABG/angioplasty (balloon)/or stent, peripheral vascular disease, diabetes, chronic kidney disease. Current prescription medications. Smoking status and alcohol intake

*Patient questionnaires - self-completion*
Health related quality of life assessed by the SF-36 [20] and EQ-5 D [19]
Disability assessed by the Modified Rankin Scale [26]
Medication Adherence Report Schedule (MARS) for BP treatment [27]
Symptoms/side effects questionnaire

*Patient questionnaire - research nurse completion*
Cognitive function assessed by the Mini Mental State [21]

*Eligibility and consent*
Review inclusion and exclusion criteria and record outcome of consent process

***Patient follow up for BP control***

**Figure 1:**
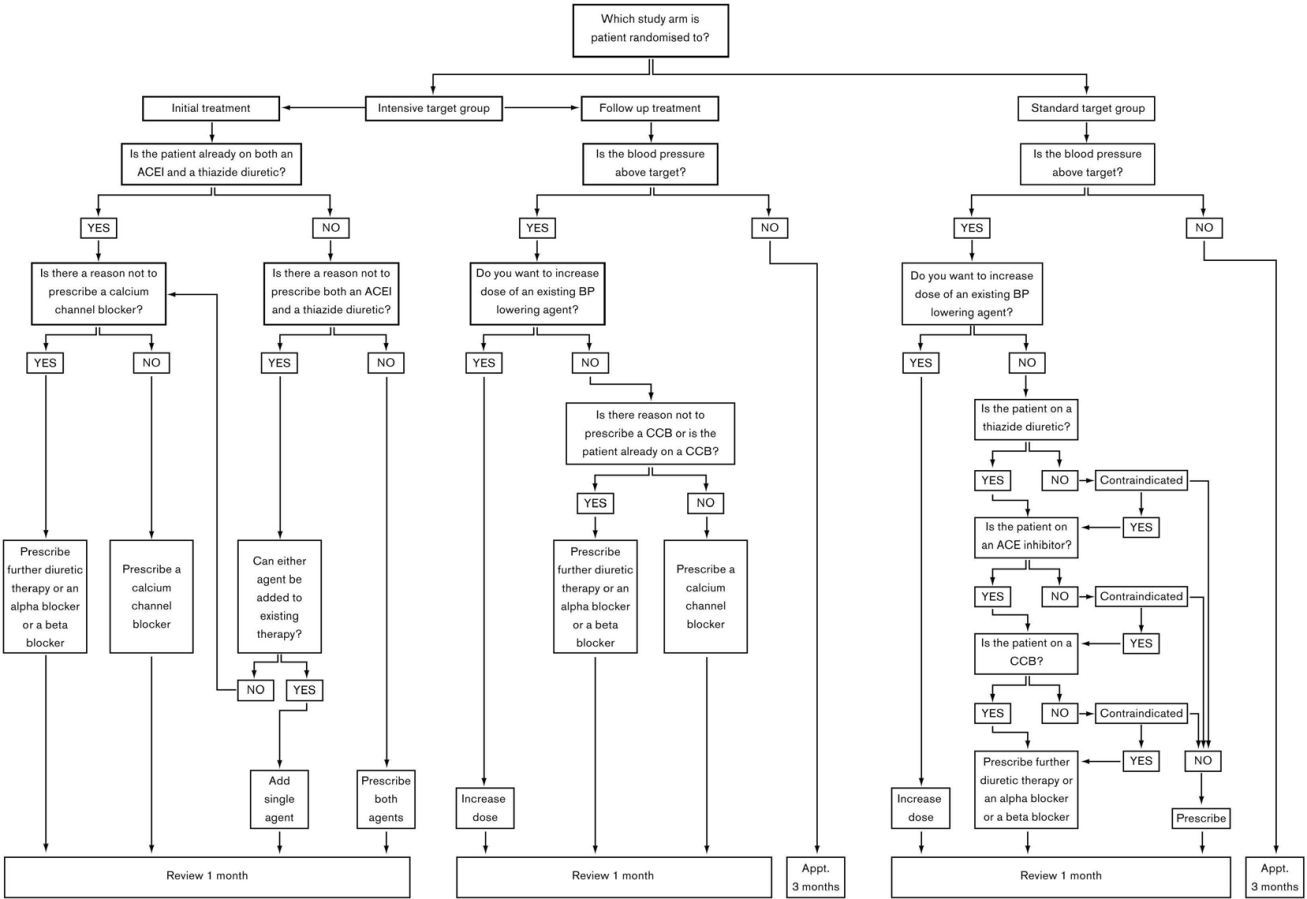
**Summary of Algorithm for BP control**.

**Figure 2:**
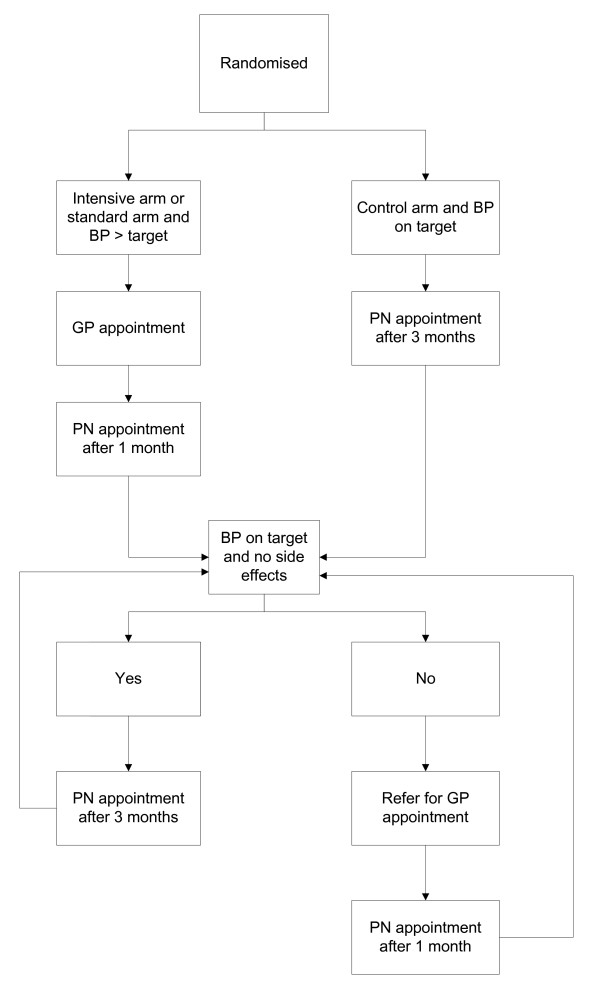
**follow up procedure**.

*GP appointment at any time patient BP medication review required*
Action taken to treat/monitor side effects
Action taken to treat BP above target using study algorithm (see figure 1)
Make appointment with practice nurse at appropriate interval (see figure 2)

*Practice nurse follow up - 1-3 month intervals*
Six BP readings, as per baseline data collection
Side effects of BP medication
Refer patient to GP or make further appointment with practice nurse (see figure 2)

***Patient follow up by research team***

*Research nurse follow up - 6 and 12 months post randomisation*
Six BP readings, as per baseline data collection
24 hour ambulatory BP recording (12 month f/u only)
Check details of patient visits to GP and practice nurse
Diagnosis of key medical conditions (as per baseline data collection) since baseline or the previous research nurse follow up
All hospital admissions or outpatient visits since baseline or the previous research nurse follow up
Record of medications introduced since baseline or the previous research nurse follow up
Monitor compliance with repeat medication since baseline or previous research nurse follow up
Completion of patient questionnaires, as per baseline data collection.

*Obtaining information on patients who died*
Records flagged at NHS central register

Once the treatment allocation has been obtained, patients in the intensive target group and any patients who have been randomised to the standard treatment group whose BP is above the target of 140 mmHg will see a GP in order to have their treatment intensified using the study specific treatment protocol (see figure 1). Patients in the standard group whose BP is below target will receive an appointment to see the practice nurse three months post randomisation.

#### Randomisation

The randomisation will use minimisation to balance the randomised groups on the basis of age (<80, ≥80), sex, diabetes mellitus, atrial fibrillation (because of the difficulties of obtaining accurate BP measurements in this group), baseline systolic BP and practice.

#### Patient follow-up procedures

Patients will be followed up from trial entry for 12 months. Follow up will be carried out in several ways. Firstly, practice nurses (PNs) will see patients at 1-3 monthly intervals, depending on BP and treatment allocation. (see figure 2) The PN will take a patient's BP and refer them to the GP if the BP is above target, or if the patient is having problems with adverse effects to their BP medication. The GP will then adjust the medication, following the study treatment protocol (see figures 1). At 6 and 12 months patients will be followed up by a research nurse (RN) where details of primary and secondary outcomes will be collected. (see table 2). Finally, the records of patients will be flagged at the NHS Central Register.

#### Patient outcome measures

The primary outcome measure is a change in systolic blood pressure between baseline and 12 months. Blood pressure measurements are performed in a standardised way, using BHS validated automated electronic monitors supplied and validated for the study. The patient will be seated for 5 minutes and then 6 measurements will be taken at minute intervals. The second and third measurements are averaged to give the reading. As the intensive target group have their BP monitored more frequently than those in the standard group, there may be some diminution of the 'white coat' effect in this group; the mean of readings 2 to 6 and the mean of 5 and 6 will be used to monitor for this. Any differences between the groups in the primary outcome should be sustained in the mean of the 5^th ^and 6^th ^readings (by which time any accommodation effect is likely to have worn off), enabling us to determine whether accommodation has a significant effect in this study. 24 hour blood pressure recordings using an ambulatory sphygmomanometer will be recorded at baseline and at twelve months. 24 hour ambulatory measurement will be unaffected by accommodation, so will provide further evidence as to whether or not this was significant in this study.

A variety of secondary outcome measures are also assessed during the RN follow up appointments. (see table 2) Key secondary outcomes are: additional measures of BP (change in diastolic and mean daytime ambulatory systolic BP between baseline and twelve months); measures of adherence (including GP adherence to protocol and patient adherence to prescribed medication); quality of life (EQ5 D [19]; SF36 [20]); side effects, tolerability and adverse events; clinical outcomes (including major cardiovascular events [composite of fatal and non-fatal stroke, myocardial infarction or fatal coronary heart disease and other cardiovascular death], all cause mortality, cognitive function [21] and hospital admissions). Key secondary events (stroke; myocardial infarction; fatal coronary heart disease and other cardiovascular death) will be reviewed by independent clinicians blinded to treatment to ensure unbiased coding of these events.

#### Sample size

Randomisation of 610 patients (305 per arm), with 12 months of follow-up, will detect a 5 mmHg difference in systolic BP between groups with 90% power and at a significance level of 5% assuming a standard deviation of 17.5 mmHg (a conservative estimate of standard deviation falling between 16, a figure derived from the same sort of patients as included in this study [11] and 19, the standard deviation in the PROGRESS Study [5]). The calculation assumes that: a 5 mmHg difference in systolic BP is of clinical significance, leading to a 20% reduction in major vascular events;[22] that there will be 5% mortality at six months, and a further 10% of patients will not have their BP measured at six months.

With regard to ambulatory blood pressure measurement, one of the secondary outcomes of the study, randomisation of 450 patients (225 per arm) will detect a 4 mmHg difference in systolic BP between groups with 90% power and at a significance level of 5% assuming a standard deviation of 11.7 mmHg [23]. This calculation assumes that 80% of patients will have ambulatory blood pressure measured at 12 months.

To recruit patients from primary care, an estimate of the number of practices is required. Approximately 50 practices with an average list size of 7,500 will be needed in order to recruit the required number of patients. This will generate 5,625 patients on practice TIA/stroke registers (from the QOF data, the overall prevalence of TIA/stroke in primary care is 1.5%). From our analysis of South Birmingham data [11], we anticipate that 13% of these patients will be ineligible because they are already on three or more anti-hypertensives, and 28% because they will not fulfil the diagnostic criteria for a history of stroke or TIA for the study [24]. We assume that 30% of patients will respond to the invitation to attend a study clinic, that 24% of them will be ineligible due to a systolic BP below 125, and a further 15% will decline to take part after discussion with the research nurse. This equates to the recruitment of 12 patients per practice with an average list size of 7,500.

### Statistical analysis

The principal analyses will use generalised linear models, accounting for baseline BP as a patient level covariate, and practices as random effects and compare differences in systolic BP (primary outcome), and differences in diastolic BP, quality of life, adherence and frequency of adverse effects (secondary outcomes). We will look at effect on systolic BP lowering in pre-specified sub-groups: diabetes; atrial fibrillation; and age group. Clinical event rates will be monitored by treatment allocation by the Data Monitoring & Ethics Committee, but only aggregated rates will be made available to the investigators.

### Economic evaluation

Decision analytical modelling will be undertaken to synthesise data from the trial and the literature in order to determine whether potential benefits of intensive blood pressure lowering (by lowering the risk of stroke) are outweighed by potential adverse effects on quality of life. Ultimately the model analyses will inform whether a further trial, powered to detect differences in clinical end-points, is required.

A Markov model will be constructed to consider intensive target and standard target strategies for blood pressure lowering in patients with a history of stroke or TIA. The clinical events of importance in the model are further stroke events, myocardial infarction (MI) and other cardiovascular related mortality. Data from the trial and literature will inform the probability of these events occurring and the risk reduction afforded by the alternative strategies. Attached to each health state will be associated health state utility values (quality of life) in order that quality-adjusted life years (QALYs) can be calculated. Quality of life on each treatment strategy will be obtained from the trial data on EQ-5 D, and previous studies will inform post-stroke and post-MI values. In addition, in order that cost-effectiveness analyses can be undertaken, the model will be populated with costs of the therapies prescribed in each strategy and acute and long term costs of further cardiovascular events.

In order to explore uncertainties in the analyses, deterministic sensitivity analysis is proposed to test the robustness of the model when varying key model parameters and structural assumptions. Probabilistic sensitivity analysis will be undertaken to incorporate the uncertainty around parameter values and quantify the overall decision uncertainty, and inform whether further research is required.

### Qualitative Study

#### Sampling

A purposively selected sample of 30 patients (10 each from intervention and control and 10 patients who declined the invitation to participate), and 20 healthcare professionals (Health Care Assistants (HCAs), nurses and GPs) will be selected for interview.

##### Patient Sampling Strategy

Sampling will be carried out on the basis of study arm (intervention or control), with a further group of people who did not consent to participate in the trial also being invited to attend for an interview. Within each group, participants will be selected on the basis of: age (tertiles); socio-economic status (using IMD scores); number of different classes of medications; and whether they have had a stroke or a TIA. A researcher will randomly select patients from these categories, ensuring that similar numbers of patients in all categories are included.

##### Health care professionals sampling strategy

Practices participating in the study were selected to ensure a range of practice characteristics are represented, including practice size and socio-economic status. A researcher will randomly select 20 practitioners from these practices and send an invitation letter and information sheet, inviting them to take part in an interview.

Patients and practitioners who fail to respond to invitation or who do not wish to participate will be replaced by another patient/practitioner with similar characteristics. This process will continue until theoretical saturation is achieved and interviews cease.

#### Interviews

Semi-structured, face-to-face, in-depth interviews will be carried out in patients' own homes or in other suitable locations, or with healthcare professionals in the surgery, and will be conducted by a researcher trained in qualitative interviewing techniques. Fully informed consent will be obtained from interviewees at the start of the interview, and a consent form signed. An interview topic guide will be used (see additional file 1) which will then be modified and refined during the first interviews. Each interview is expected to last between 60 and 90 minutes, and will be audio taped and transcribed verbatim.

#### Data Analysis

Data collection and analysis will be iterative, occurring as data collection in the interviews proceeds. Data will be analysed using a thematic approach, based on the principles of 'Framework' analysis [25] and using Framework software. The research team will actively contribute to the development of the analysis and conceptual framework and their different disciplinary and professional backgrounds will maximise theoretical sensitivity [16].

#### Time plan

Patient recruitment began in July 2009 and is planned to continue until February 2011. By October 2009, 23 patients (4% of target) have been recruited into the trial. Interviews will commence in January 2010 and are expected to be completed by February 2011.

## Discussion

The results of this trial and the health economic analysis will provide insight into the role of intensive blood pressure targets for people who have had a stroke or TIA. If the trial is negative and a significant difference in systolic blood pressure is not observed between the two study arms, then the embedded qualitative work will be of importance to determine why low blood pressure targets did not lead to lower blood pressure. If the trial is positive, then the critical question remains as to whether striving for lower blood pressure targets is appropriate. If we observe no difference in adverse event rates or quality of life between the two arms of the trial, then it is likely that aiming for lower blood pressure targets will be worthwhile, given the benefits of reduced stroke risk that were observed in the PROGRESS trial [5]. This will be tested by our economic analysis. If, on the other hand, the lower blood pressures are at the cost of higher adverse event rates, then it may be that a further trial powered to detect differences in clinical end-points will be required to guide clinical practice.

## Competing interests

GF or his institution has received payment for educational, advisory and research activities related to BP lowering drugs in the last 12 months from the following pharmaceutical companies: Boehringer Ingelheim, Servier.

FDRH has received limited research support in terms of BP devices from Microlife and BpTRU and occasional sponsorship or speaker fees from a number of pharmaceutical companies that market anti-hypertensives.

RJM is funded by an NIHR Career Development Fellowship and has no conflicts with respect to this paper.

NF has received funding for research and consulting from several companies who manufacture treatments for cardiovascular disease.

None of the remaining authors have any conflicts of interest.

## Authors' contributions

JM conceived the study. JM, KF, SC, GF, RH, RM and UM designed the RCT; KF, SV and SG were responsible for the design of the qualitative element; SJ, JM, CT and RM participated in the design of the health economics; JB, CT, and JM designed the treatment algorithm and NF participated in the design of the statistics. KF drafted the manuscript and all authors contributed to its revision. All authors have given final approval of the version to be published. JM is the study guarantor.

## Pre-publication history

The pre-publication history for this paper can be accessed here:

http://www.biomedcentral.com/1471-2261/10/37/prepub

## Supplementary Material

Additional file 1 **Interview guides**. This file contains a copy of the interview guides for Patients and Health Care Professionals. Click here for file
