# Supplementary material for: Protocol for Past BP: a randomised controlled trial of different blood pressure targets for people with a history of stroke of transient ischaemic attack (TIA) in primary care
Source: BMC Cardiovasc Disord. 2010 Aug 9;10:37. doi: 10.1186/1471-2261-10-37 (PMC2923098; doi:10.1186/1471-2261-10-37)
Supplement: Additional file 1 — Interview guides. This file contains a copy of the interview guides for Patients and Health Care Professionals. [file 1471-2261-10-37-S1.DOC]

Interview Guides

| ***Patient Interview Guide – RCT Patients*** |
| --- |
| We are carrying out some research to try and find out people’s views about high blood pressure and stroke and the ways in which these can be treated. |
| *Patient details*  If we could just begin by confirming some personal details:  What is your date of birth?  How would you describe your ethnicity?  What is your highest educational qualification? |
| *Patient understanding/attitudes towards medication.*  Are you currently taking any tablets for your BP?  Do you know why the GP has put you on these tablets?  Did the GP explain the reasons to you? What did the GP say was the most important reason?  How did you feel about the level of explanation? Was enough explanation provided? Could the explanation have been better? If so, how?  Were you given any additional information about your tablets? (e.g. written information)  Would you have liked more information? In what way?  Overall, did you feel that you were well-informed about the tablets and why you were being given them?  How did you feel about the decision to start medication?  Did you have any involvement in making that decision? Would you have liked more involvement?  How long have you been taking your tablets?  Do you take your tablets every day?  Do you ever miss a dose? What do you do when this happens?  Do you think it is important to take these tablets? (Explore reasons why)  What do you think would happen if you didn’t take your tablets?  How have you got on with the tablets? Are they easy to take?  Have you had any problems with the tablets? (e.g. size of the tablet, too many tablets, side effects from the tablets) If so, have you discussed any of these problems with your GP?  Have there been any benefits from taking the tablets?  Do these benefits outweigh the problems?  Do you do anything else to help lower you BP? (For example, exercise, altering diet). Do you think it has helped? |
| *Patient attitude/understanding towards BP measurement.*  Do you think it is important to have your BP measured? (Explore reasons why)  What are the benefits of having your BP measured?  How often do you have to attend the practice to have your BP measured?  How convenient is it to attend?  Have you had any problems getting to the practice?  How do you feel about the current frequency with which your BP is being measured? Is it about right? Would you like it to be measured more/less often?  How do you feel about the service you receive when you have your blood pressure measured? |
| *Experience of taking part in the RCT (randomised patients only)*  Why did you decide to take part in the trial?  Did you understand what the trial would involve before you began?  Did you feel that the trial was explained clearly to you?  Was taking part in the trial what you had expected?  What was your understanding of BP before taking part in the trial? Has taking part in the trial improved your understanding?  Have there been any benefits from taking part in the trial?  Have there been any draw-backs/problems from taking part in the trial?  Have you enjoyed taking part in the trial? What have you enjoyed? Is there anything you have not enjoyed?  Would you take part in the trial again? (Explore reasons). If so, are there any changes/improvements you would like to see? |
| *Health care professional ( HCP) Interview Guide* |
| We are carrying out some research to try and find out health care professionals views towards blood pressure. |
| *HCP understanding and attitudes towards BP, medication and measurement*  How do you explain to patients that they have high BP and that they need to go onto medication?  How well do you think your patients understand this?  How well do you think they understand the importance of taking their medication regularly?  When the decision is made to start a patient on treatment, how involved is the patient in the decision making?  Do you think there are some patient groups that are easier or more difficult to treat? (For e.g. older or male patients). If so, which groups? (Explore reasons why).  What do you think about trying to set a more intensive BP target for people with stroke or TIA? Is it a good/bad idea? (Explore reasons)  Do you think attempts to achieve lower or more intensive BP targets realistic?  How easy is it to justify lower BP targets to patients?  Do you think patients have experienced additional side-effects from trying to achieve a lower BP target?  Do you think the trade off of achieving a lowering BP target for patients, is worth the benefits?  If you were prescribed BP lowering medication, would you want to take it?  How often would you want to be monitored?  Would you still take the medication if you suffered from side effects? |
| *Experience of taking part in the RCT*  Why did your practice decide to take part in the trial?  Was taking part in the trial what you had expected?  How well do you think patients in the trial understand the trial? Are there are patient groups who find it difficult to understand the trial? For example, older patients.  How realistic has the lower BP target of 130 systolic (or a 10mmHg reduction if the baseline systolic is below 140mmHg) for patients in the interventional group been?  If you were a patient, would you take part in the trial? (Explore reasons)  Are there any implications for you or the practice concerning the intensive targets? (For example QOF quotas, workload, cost)  Has your practice enjoyed taking part of the trial? What parts of the trial in particular have the practice enjoyed? (Explore reasons)  Would your practice take part in the trial again? (Explore reasons). If so, are there any changes/improvements you would like to see? |
